# Logic Learning Machine creates explicit and stable rules stratifying neuroblastoma patients

**DOI:** 10.1186/1471-2105-14-S7-S12

**Published:** 2013-04-22

**Authors:** Davide Cangelosi, Fabiola Blengio, Rogier Versteeg, Angelika Eggert, Alberto Garaventa, Claudio Gambini, Massimo Conte, Alessandra Eva, Marco Muselli, Luigi Varesio

**Affiliations:** 1 Laboratory of Molecular Biology, Gaslini Institute, Largo Gaslini 5, 16147 Genoa, Italy; 2 Institute of Electronics, Computer and Telecommunication Engineering, National Research Council of Italy, Genoa 16149, Italy; 3 Department of Human Genetics, Academic Medical Center, University of Amsterdam, Meibergdreef 15, Amsterdam 1100, The Netherlands; 4 Department of Pediatric Oncology and Hematology, University Children's Hospital Essen, Hufelandstr. 55, Essen 45122, Germany; 5 Department of Hematology-Oncology, Gaslini Institute, Largo Gaslini 5, Genoa 16147, Italy; 6 Departments of Pediatric Pathology, Gaslini Institute, Largo Gaslini 5, Genoa 16147, Italy

## Abstract

**Background:**

Neuroblastoma is the most common pediatric solid tumor. About fifty percent of high risk patients die despite treatment making the exploration of new and more effective strategies for improving stratification mandatory. Hypoxia is a condition of low oxygen tension occurring in poorly vascularized areas of the tumor associated with poor prognosis. We had previously defined a robust gene expression signature measuring the hypoxic component of neuroblastoma tumors (NB-hypo) which is a molecular risk factor. We wanted to develop a prognostic classifier of neuroblastoma patients' outcome blending existing knowledge on clinical and molecular risk factors with the prognostic NB-hypo signature. Furthermore, we were interested in classifiers outputting explicit rules that could be easily translated into the clinical setting.

**Results:**

Shadow Clustering (SC) technique, which leads to final models called Logic Learning Machine (LLM), exhibits a good accuracy and promises to fulfill the aims of the work. We utilized this algorithm to classify NB-patients on the bases of the following risk factors: Age at diagnosis, INSS stage, MYCN amplification and NB-hypo. The algorithm generated explicit classification rules in good agreement with existing clinical knowledge. Through an iterative procedure we identified and removed from the dataset those examples which caused instability in the rules. This workflow generated a stable classifier very accurate in predicting good and poor outcome patients. The good performance of the classifier was validated in an independent dataset. NB-hypo was an important component of the rules with a strength similar to that of tumor staging.

**Conclusions:**

The novelty of our work is to identify stability, explicit rules and blending of molecular and clinical risk factors as the key features to generate classification rules for NB patients to be conveyed to the clinic and to be used to design new therapies. We derived, through LLM, a set of four stable rules identifying a new class of poor outcome patients that could benefit from new therapies potentially targeting tumor hypoxia or its consequences.

## Background

Neuroblastoma is the most common solid pediatric tumor, deriving from ganglionic lineage precursors of the sympathetic nervous system [1]. It shows notable heterogeneity of clinical behavior, ranging from rapid progression associated with metastatic spread and poor clinical outcome to spontaneous, or therapy-induced, regression into benign ganglioneuroma. Age at diagnosis, stage and amplification of the N-myc proto-oncogene (*MYCN*) are clinical and molecular risk factors that the International Neuroblastoma Risk Group (INRG) utilized to classify patients into high, intermediate and low risk subgroups on which current therapeutic strategy is based. About fifty percent of high risk patients die despite treatment making the exploration of new and more effective strategies for improving stratification mandatory [2].

The availability of genomic profiles improved our prognostic ability in many types of cancers [3]. Several groups used gene expression-based approaches to stratify neuroblastoma patients. Prognostic gene signatures were described [4-10] and classifiers were trained to predict the risk class and/or patients' outcome [4-11]. It was recently reported the design of a multi signature ensemble classifier that merges heterogeneous, neuroblastoma-related gene signatures to blend their discriminating power, rather than numeric values, into a single, highly accurate patients' outcome predictor [12]. However, it is difficult to interpret these results with respect to the underlying biology because the assembly of the signature is only computational. The translation of the computational results to the clinic requires the use of explicit statements, coupled with the capability of blending prior knowledge on the disease with newly acquired information from high throughput technology.

We developed a biology-driven approach which defines the gene expression profile of a biological process known to be relevant, by prior knowledge, to the progression of the tumor and we then evaluate the prognostic value of such signature. We have identified tumor hypoxia as a feature of highly aggressive neuroblastoma [13]. Hypoxia is a condition of low oxygen tension occurring in poorly vascularized areas of the tumor which has profound effects on cell growth, genotype selection, susceptibility to apoptosis, resistance to radio- and chemotherapy, tumor angiogenesis, epithelial to mesenchymal transition and propagation of cancer stem cells [14-17]. Hypoxia activates specific genes encoding angiogenic, metabolic and metastatic factors [15,18] and contributes to the acquisition of the tumor aggressive phenotype [15,19,20]. We have used gene expression profile to assess the hypoxic status of neuroblastoma cells and we have derived a robust 62-probe sets neuroblastoma hypoxia signature (NB-hypo) [13,21] which is an independent risk factor for neuroblastoma patients [22]. Prognostic signatures that can be linked to a biological processes are of great interest because they may redirect the choice of drugs in the design of more effectives treatments. Therefore, integration of the NB-hypo signature with the existing risk factors may result into an interesting, needed and improved tool for neuroblastoma patients stratification and treatment.

Several statistical and machine learning techniques have been proposed in the literature to deal with output of explicit rules and good classification performance [23]. Most available techniques, such as linear discriminant approaches, multilayer perceptrons or support vector machines, are able to achieve a good degree of provisional accuracy, but construct black-box model whose functioning cannot allow to derive information about the pathology of interest and its relationships with the considered diagnostic and prognostic factors. To overcome this drawback, different classification methods, capable of constructing models described by a set of intelligible rules, have been developed. Methods based on Boolean Function Synthesis adopt the aggregative policy according to which at any iteration some patterns belonging to the same output class are clustered to produce an intelligible rule. Suitable heuristic algorithms [24-26] are employed to generate rules exhibiting the highest covering and the lowest error; a tradeoff between these two different objectives has been obtained by applying the Shadow Clustering (SC) technique [24] which generally leads to final models, called Logic Learning Machines (LLM), exhibiting a good accuracy.

In the present work, we describe the utilization of LLM to generate rules classifying NB patients on the basis of NB-hypo and clinical and molecular risk factors. We found that this algorithm can generate rules stratifying high risk neuroblastoma patients who could benefit from new therapeutic approach related to hypoxia. Finally, we introduce a workflow to identify the instances that generate rules instability and to generate rules with a high degree of stability.

## Results

Our aim was to derive explicit and stable rules, based on risk factors, to stratify NB patient's outcome. Initially, we studied a 182 NB patients dataset characterized by three common risk factors, Age at diagnosis, INSS stage, MYCN oncogene amplification. "Good" or "poor" outcome classes are defined, from here on, as the patient's status "alive" or "dead" 5 years after diagnosis, respectively (Table 1). The composition of the dataset is comparable to what previously described [5]. We selected this dataset because the gene expression profile of the primary tumor, performed by microarray, was available for each patient. We applied the LLM implemented by the Rulex 2.0 software to generate intelligible rules predicting patients' outcome. The results are shown in Table 2. The algorithm identified 5 explicit rules predicting outcome. Each rule covered more than one patient. In fact, the total covering of patients predicted dead (rules 2.1 and 2.2) adds up to 140% but it comprises 96% of non overlapping poor outcome patients. Rules 2.3, 2.4 and 2.5 include 87.1% non overlapping good outcome patients. Classification error was calculated for each rule as detailed in the Methods section. In general, the prediction error was quite low with the exception of rule 2.2 which had 13% error. This high error was somewhat predictable because the rule applies to stage 4 patients known to be resilient to classification by common risk factors [5]. Rule 2.5 is in total agreement with the known good prognosis of patients diagnosed before one year of age [27]. We then assessed the concordance of the predictions with the stratification in Low (LR), Intermediate (IR) and High risk classes (HR) proposed by the INRG on the same risk factors. HR patients were correctly included in the rules classifying poor outcome patients and LR patients mapped in the good outcome rules. The concordance between risk prediction by the rules and previous clinical knowledge provided the first indications of the suitability of LLM to classify neuroblastoma patients.

**Table 1:** Characteristics of the neuroblastoma patients datasets

**Risk factors**		**Training set^a^**		**Independent test set^b^**	
		**Patients**	**Distribution (%)^C^**	**Patients**	**Distribution (%)^C^**
**Age at diagnosis (Years)**					
	<1	86	47	26	51
	≥1	96	53	25	49
**INSS stage**					
	1	42	23	13	25
	2	24	13	7	14
	3	23	13	11	21
	4	69	38	16	32
	4s	24	13	4	8
**MYCN status**					
	normal	152	84	40	79
	amplified	30	16	11	21
**Outcome**					
	Good	131	72	41	80
	Poor	51	28	10	20
**Risk Group ^d^**					
	LR	94	52	27	53
	IR	21	11	8	15
	HR	67	37	16	32

^a ^The total number of patients is 182. The number of patients in each subdivision is shown.

^b ^The total number of patients is 51. The number of patients in each subdivision is shown.

^c ^Percentage relative to the total number of patients.

^d ^The risk assessment is based on INRG classification. HR = High Risk; IR = Intermediate Risk; LR = Low Risk.

**Table 2:** Classification rules predicting neuroblastoma patients' outcome on the bases of INRG risk factors

**Rule ID^a^**		**INSS stage**	**MYCN status**	**Age at diagnosis (Years)**		**Predicted Outcome**	**Covering ^b ^(%)**	**Error ^c ^(%)**	**Risk group ^d^**
**2.1**	*IF (*	{1, 3, 4, 4s}	amplified	≥1	*)THEN*	Poor	50	1.5	HR
**2.2**	*IF (*	{4}	_	≥1	*)THEN*	Poor	90	13	HR
**2.3**	*IF (*	{1, 2, 4s}	_	<1	*)THEN*	Good	68	1.9	LR
**2.4**	*IF (*	{1,2,3,4s}	normal	_	*)THEN*	Good	81	3.9	LR
**2.5**	*IF (*	_	_	<1	*)THEN*	Good	65	0	LR,IR

^a ^The Rule ID is composed by the table number followed by a dot and the rule number.

^b ^Covering is the fraction of examples in the training set that verify the rule and belong to the target class.

^C ^Error is the fraction of examples in the training set that satisfy the rule and do not belong to the target class.

^d ^The risk assessment is based on INRG classification taking into consideration only INSS stage, MYCN status and Age at diagnosis. HR = High Risk; IR = Intermediate Risk; LR = Low Risk.

Fifty one patients failed to respond to treatment and have poor prognosis (Table 1) indicating the need to develop and test new risk factors to improve stratification and new therapeutic protocols. Biology-driven gene expression signatures have the potential to fulfill the dual purpose of generating new attributes for patients stratification and to indicate the possible therapeutic strategy [22]. We previously described a 62-probe sets signature (NB-hypo) that stratifies neuroblastoma patients on the bases of outcome [22]. We addressed the effects of the addition of NB-hypo as risk factor on outcome prediction. Rulex utilizes only categorical attributes. We divided the patients in groups through the application of k-means clustering of the patients based on the 62-probe sets signature of NB-hypo. Two was the optimal number of clusters to partition the dataset as shown by the within cluster distance when varying the number of clusters (see additional Figure 1 in Additional file 1). The 182 patients were clustered in two groups of 136 and 46 elements with Low and High NB-hypo expression respectively (see additional Figure 2 in Additional file 1).

We tested the effect on classification of including NB-hypo to the previously analyzed INRG risk factors and the results are shown in Table 3. The algorithm produced a 7 rules classifier of which rules 3.1, 3.2 and 3.3 cover 76.4% of non overlapping poor outcome patients and rules 3.4, 3.5, 3.6, and 3.7 cover 95.4% of non overlapping good outcome patients. We called this classifier Initial classifier. Each rule utilizes some, but not all, considered risk factors and NB-hypo was included in three rules demonstrating its potential in NB stratification. The rules classifying poor outcome patients have a very low error ranging from 1.5% to 3% indicating a good performance of the algorithm in predicting this class. Furthermore, rules 3.1 and 3.2 include patients with stage 4 neuroblastoma, a group that is traditionally very difficult to be stratified, demonstrating that NB-hypo is an important prognostic factor for high risk patients. In contrast, the classification of good outcome patients produced rules with variable error, including rule 3.5 with 23% error. The statistical significance of each rule was assessed by Fisher exact test demonstrating a high significance of every rule with the exception of rule 3.7 that had also minimal covering. Finally, we introduced a new parameter, the stability value, which provides an indication of the robustness of the rules. The stability of the rules was somewhat variable ranging from 0.50 of rule 3.3 to 0.94 and we addressed this issue in detail (see below). In conclusion, we demonstrated that NB-hypo could be successfully utilized to generate rules stratifying NB patients. Interestingly, the prognosis of stage 4 patients took advantage of NB-hypo providing the first evidence of the importance of this new risk factor in stratifying high risk patients.

**Table 3:** Classification rules of neuroblastoma patients including NB-hypo

**Rule ID^a^**		**NB-hypo**	**INSS Stage**	**MYCN Status**	**Age at diagnosis (Years)**		**Predicted Outcome**	**Covering ^b ^(%)**	**Errore^C ^(%)**	**Fisher pvalue^d^**	**Stability^e^**
**3.1**	*IF (*	High	{3,4}	_	≥1	) *THEN*	Poor	64	3	<0.001	0.94
**3.2**	*IF (*	High	{2,3,4}	Normal	≥1	) *THEN*	Poor	25	2.2	<0.001	0.8
**3.3**	*IF (*	_	{1, 3, 4, 4s}	Amplified	≥1	) *THEN*	Poor	50	1.5	<0.001	0.5
**3.4**	*IF (*	_	_	_	<1	) *THEN*	Good	65	0	<0.001	0.94
**3.5**	*IF (*	Low	_	Normal	_	) *THEN*	Good	89	23	<0.001	0.64
**3.6**	*IF (*	_	{1, 4s}	_	_	) *THEN*	Good	50	0	<0.001	0.9
**3.7**	*IF (*	_	{1, 2, 4s}	Amplified	_	) *THEN*	Good	1.5	0	>0.5	0.8

^a ^The Rule ID is composed by the table number followed by a dot and the rule number.

^b ^The covering is the fraction of examples in the training set that verify the rule and belong to the target class.

^C ^The error is the fraction of examples in the training set that satisfy the rule and do not belong to the target class.

^d ^Fisher p-value quantifies the statistical significance of the rule.

^e ^Stability measures the fraction of the occurrences of a given rule in a 5 rounds of 10 fold cross validations.

**Figure 1:**
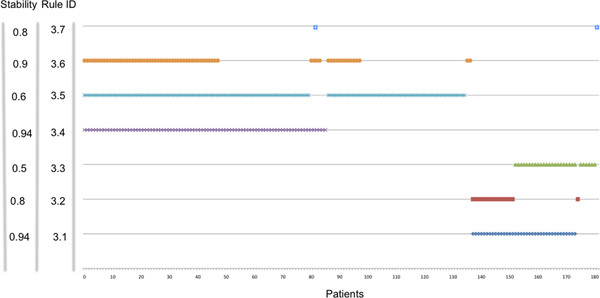
**Patients representation in the rules of Table 3**. Plot of the membership of the 182 patients (x axis) to the rules of the classifier in Table 3. Each patient in our dataset is covered by at least one rule.

**Figure 2:**
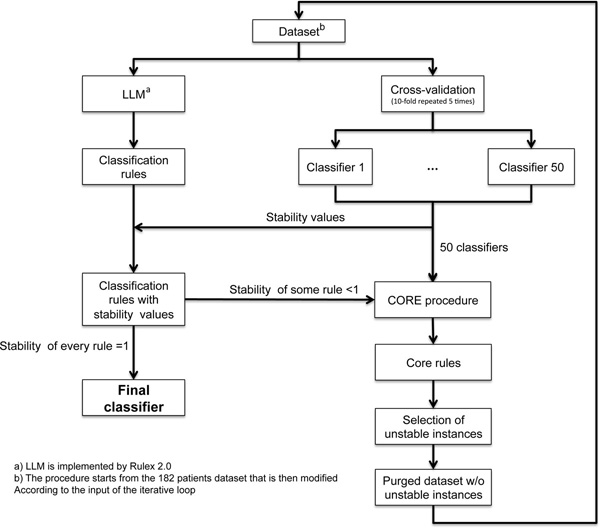
**Rules stabilization flowchart**. The initial dataset is analyzed by LLM to generate the initial classifier and the stability of the rules is assessed in cross validation as described in the Methods section. The stability of each rule is associated to the classifier which is accepted if the stability of every rule is equal to one. If this condition does not apply, Core rules will be derived from the 50 classifiers originated by the cross validation procedure. These rules allow to identify the instances that generate instability which are deleted from the dataset. The purged dataset serves as input of a new cycle of analysis which is repeated till each rule has stability equal to one.

**Figure 3:**
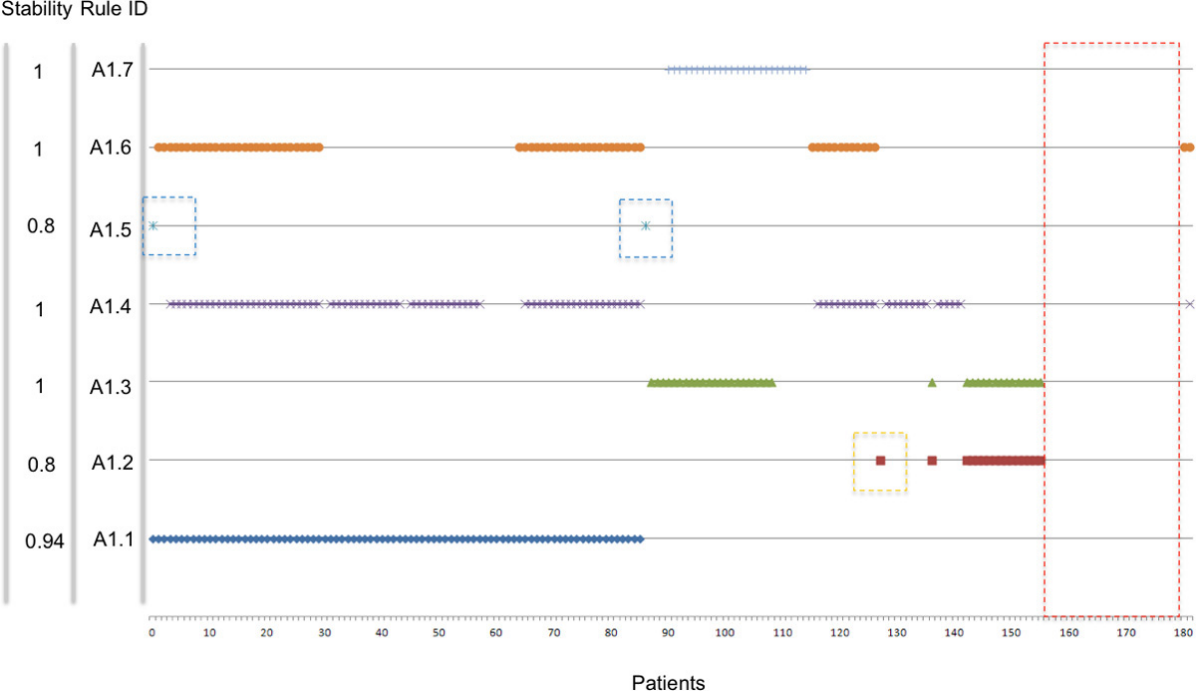
**Core rules representing the initial set of 182 patients**. Plot of the membership of the 182 patients (x axis) to the rules of the classifier in Additional file 2 Table 1. Red dashed box highlights the group of patients not covered by any Core Rule. Blue dashed box highlights the only two patients covered by rule A1.5. Orange dashed box highlights the only patients that forces the existence of rule A1.2 that would be otherwise part of A1.3.

The impact of the LLM rules on the clinic is directly related to their stability. Our results indicated a variation in stability that impacted on the "credibility" of the rules and we decided to investigate further the source of variability and, possibly, to improve the reproducibility of the rules in cross validation. Rulex 2.0 is programmed to include every patients in at least one rule as shown in Figure 1 thereby creating rules with very limited coverage, like rule 3.7, and potentially highly unstable. We utilized the stability statistical measure and the CORE procedure to identify and eliminate the instances responsible for rule instability according to the stabilization flowchart outlined in Figure 2. The process consists in an iterative procedure that removes the instances that generate instability. The process is repeated till each rule has the maximal stability value of one. The fist iteration applied to the initial 182 patients generated seven Core rules (see additional Table 1 in Additional file 2). Figure 3 shows the representation of the individual patients in the Core rules. The stability of the Core rules was clearly improved and four of them reached the one value. The major change caused by the introduction of Core rules was that 24 patients (red dotted box) were not covered by any rule. These patients shared the same characteristics (NB-hypo = Low, INSS stage = 4, MYCN status = normal and Age at diagnosis ≥ 1 year) but contained equal proportion of good and poor prognosis patients. It follows that these patients are not classifiable and their inclusion in either class depends only on the casual imbalance in the distribution between training and test set. Another source of instability is the rule A1.5 (blue dotted boxes) with only two patients whose casual association with training or test set would decide the prediction. The last case of instability regards rule A1.2 (orange dotted box), that is almost equivalent to rule A1.3 with the exception of one instance that is present only in the rule A1.2. In particular, being A1.2 equivalent to rule 3.2 and A1.3 equivalent to the rule 3.1, Rulex generates a rule 3.2 only when that unique example patient that is not covered by rule 3.1 is present in the training set.

**Figure 4:**
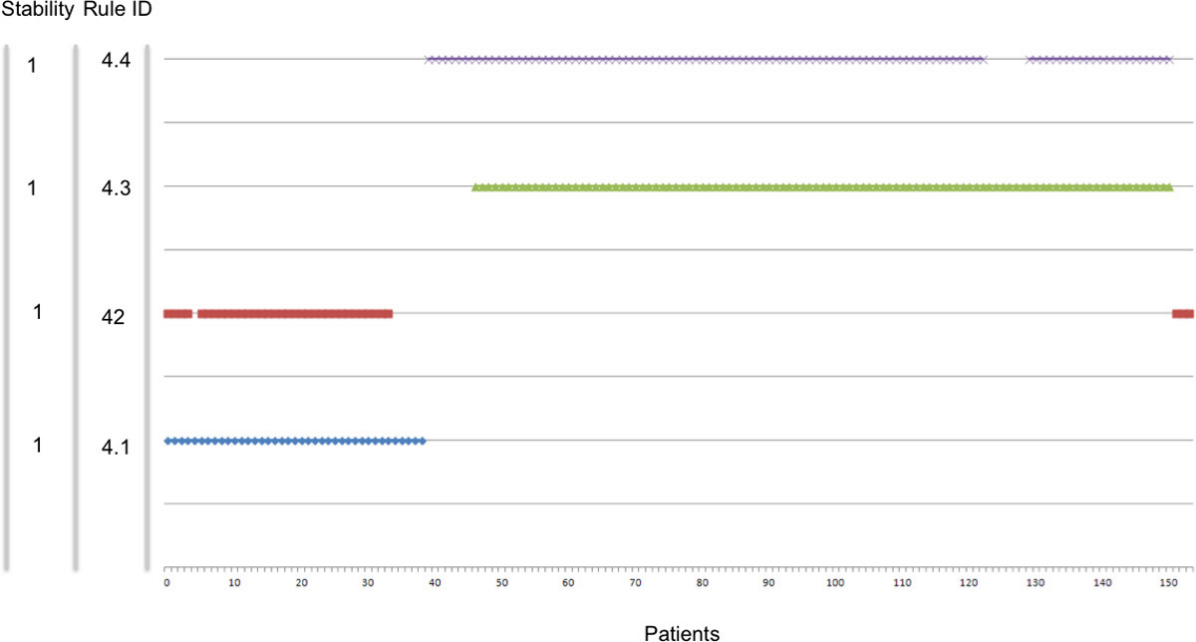
**Final classifier**. Plot of the membership of the 154 patients remaining in the dataset after purge (x axis) to the rules of the classifier in Table 4 (y axis). The stability of each rule is listed along the y axis.

**Figure 5:**
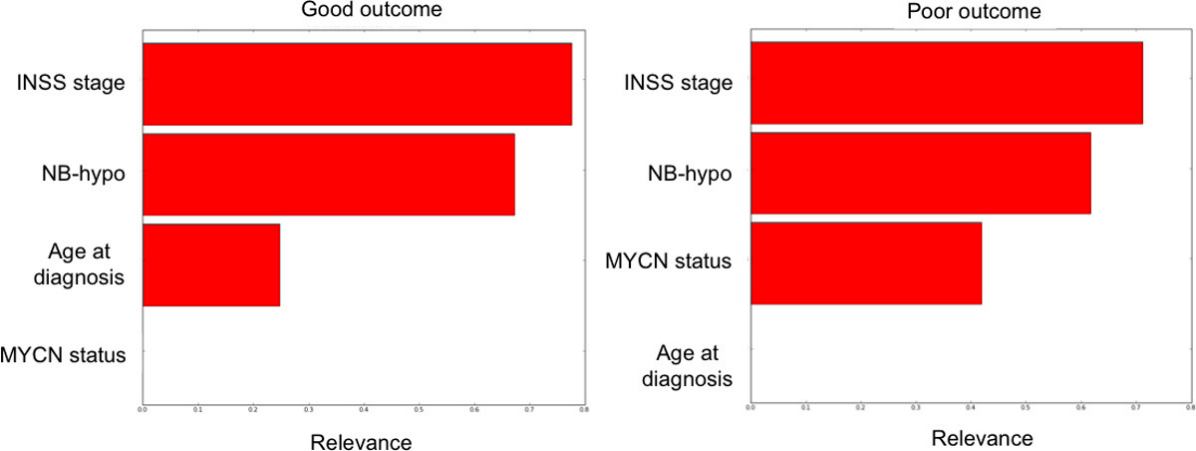
**Importance of the risk factors present in the Final classifier**. The relative relevance of the risk factors utilized by the rules utilized by the Final classifier is shown. The risk factors are INSS stage, NB-hypo, age at diagnosis and MYCN status. Right panel shows the determinations of Poor outcome patients and the left panel that of Good outcome patients the algorithms to generate the relevance values is detailed in the Methods section.

The above mentioned 27 patients were removed from the dataset as source of instability and a second round of stabilization (Figure 2) was started on the remaining 155 instances. The second iteration generated the second classifier having three out of five rules with a stability equal to one (see additional Table 2 in Additional file 2) and five Core rules in which only one had a stability smaller than one (see additional Table 3 in Additional file 2). We identified and removed the patient that was the cause of this instability. In the third interaction we obtained the Final classifier consisting of four rules each with the stability of one and lacking isolated instances (Figure 4). In conclusion, we identified and removed 28 patients causing instability corresponding to 15% of the dataset. The characteristics of the Final classifier are shown in Table 4. The statistical significance of each rule is less than 0.001 and the error is less than 4% (0% in two rules). Rules 4.1 and 4.2 cover 100% of non overlapping poor outcome patients and rules 4.3 and 4.4 cover 96% of non overlapping good outcome patients. Rule 4.2 is important because it classifies stage 4 tumors, and predicts poor outcome when NB-hypo is High. Moreover, Low NB-hypo is involved in predicting good prognosis of NB patients (Rule 4.3). We list the INRG risk groups of the patients included in the final rules based on age, MYCN and stage. Interestingly, HR patients are covered by distinct rules supporting the conclusion that NB-hypo allows the identification of new risk groups among the NB patients. The relative relevance of the risk factors included in the rules for the classification was computed and the results are shown in Figure 5. Relevance for Good outcome patients was INSS stage 0.71, NB-hypo 0.61, MYCN status 0.41, Age at diagnosis 0.0; the relevance for Poor outcome patients was INSS 0.77, NB-hypo 0.67, Age 0.24, MYCN 0.0. The relevances of NB-hypo and INSS stage were very close but far from the other risk factors providing evidence of the relevance of NB-hypo in patients'classification.

**Table 4:** Final classifier

**Rule ID ^a^**		**NB-hypo**	**INSS Stage**	**MYCN Status**	**Age at diagnosis (Years)**		**Predicted Outcome**	**Covering b (%)**	**Error ^C^** **(%)**	**Fisher pvalue^d^**	**Stabilty^e^**	**Risk group ^f^**
**4.1**	*IF (*	_	{4}	_	≥1	) *THEN*	Poor	91	4	<0.001	1	HR
**4.2**	*IF (*	High	{2, 3, 4}	_	≥1	) *THEN*	Poor	86	3	<0.001	1	HR,IR,LR
**4.3**	*IF (*	Low	_	Normal	_	) *THEN*	Good	89	0	<0.001	1	LR,IR
**4.4**	*IF (*	_	{1 2 3 4s}	Normal	_	) *THEN*	Good	90	0	<0.001	1	LR IR

^a ^The rule ID is composed by the table number followed by a dot and the rule number.

^b ^Covering is the fraction of examples in the training set that verify the rule and belong to the target class.

^C ^Error is the fraction of examples in the training set that satisfy the rule and do not belong to the target class.

^d ^Fisher p-value quantifies the statistical significance of a rule.

^e ^Stability is the fraction of the occurrences of a given rule in a 5 rounds of 10 folds cross validations.

^f ^The risk assessment is based on INRG classification taking into consideration only INSS stage, MYCN status and Age at diagnosis. HR = High Risk; IR = Intermediate Risk; LR = Low Risk.

The last task was to test the Final Classifier *g*(***x***) for conflicts. according to the procedure indicated in the Material and Methods Section. We identified potentially conflicting rules in the following pairs of rules: 4.1 vs 4.3 and 4.2 vs 4.4. In fact, the pattern ***x ***having *NB-hypo *= *Low, INSS *= 4, *MYCN *= *normal*, and *Age *≥ 1 verifies rules 4.1 and 4.3 with opposite predictions. Similarly, pattern with *NB-hypo *= *High, INSS *∈ {2,3}, *MYCN *= *normal*, and *Age *≥ 1 satisfies the conditions rules 4.2 and 4.4, with opposite predictions. However, such instances do not occur as it can be determine by the data in Figure 4 that demonstrate the lack of any overlapping among patients included in the rules predicting good (Rules 4.3 and 4.4) and poor (Rules 4.1 and 4.2) outcome. There are no patients in a conflicting situation is that because such cases were removed by the stabilization procedure as causes of instability. The potentially conflicting situations could also be computed by the rules:

**if ***NB-hypo *= *Low ***and ***INSS *= 4 **and ***MYCN *= *normal ***and ***Age *≥ 1 **then ***NC*

**if ***NB-hypo *= *High ***and ***INSS *∈ {2,3} **and ***MYCN *= *normal ***and ***Age *≥ 1 **then ***NC*

generated as described in the Material and Methods section. However, the use of the stabilization procedure is superior because it identifies correctly not only 26 patients in a conflicting situation but also 2 additional instances causing instability.

**Figure 6:**
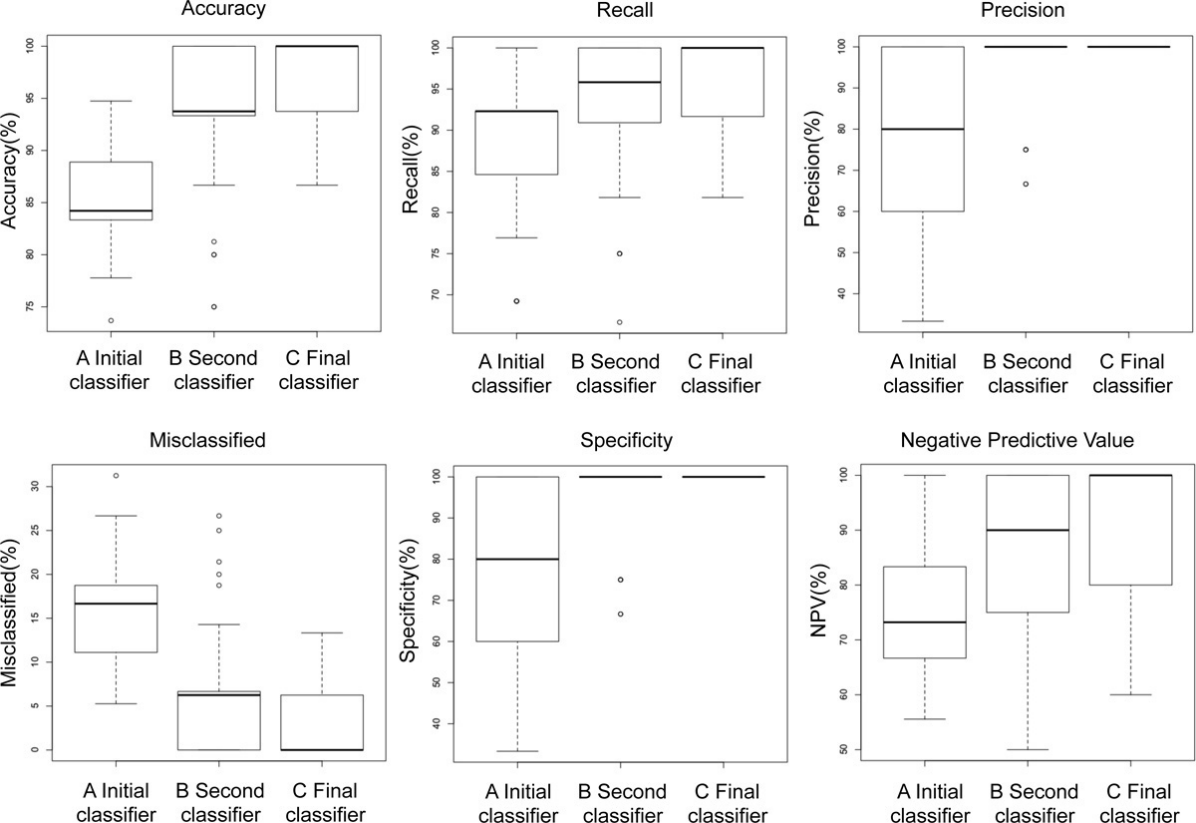
**Classification parameters and stability of the rules**. The panels of figure show accuracy, recall, precision, misclassified, specificity and negative predictive value of the initial classifier based on 182 patients (A) the second classifier based on 155 patients (B) and the Final classifier consisting of absolutely stable rules (C).

The process of stabilization of the rules is associated with an improvement of the classification parameters. Figure 6 shows a comparison of accuracy, recall, precision, specificity and negative predictive value among the Initial classifier generated on the whole 182 patients dataset, the Second classifier obtained after two cycles of stabilization and the Final classifier. Every indicator of the classifier performance improved concomitantly with the strengthening of the rules' stability with the exception of the proportion of misclassified that decreased to null values as expected by the nature of the stabilization workflow (Figure 2).

10 fold cross validation is a powerful indicator of the performance of our classifier in the 182 patients' dataset. However, the stabilization workflow called for removal of patients from the dataset raising the question of the performance of the algorithm on an independent dataset of neuroblastoma patients. We tested the predictions of the Final classifier (Table 4) on an independent 51 patients' dataset (Table 1). We deleted from the 51 patients' dataset 8 instances (15%) with the same characteristics (attribute values) as those eliminated from the 182 dataset because source of instability. The performance of the Final classifier was then tested on the remaining 43 instances. The results are shown in Table 5. The Final classifier demonstrated a good performance on the validation set resulting in 86% accuracy, 91% recall, 90% precision and 70% specificity and negative predictive value demonstrating the validity of our approach and its reproducibility on an independent dataset. These statistical measures of the performance are comparable, but somewhat lower to those obtained in cross validation (Figure 6).

**Table 5:** Performance comparison between Final, INRG risk factors and INRG consensus pretreatment schema classifiers

**Classifier**	**Accuracy ^a^**	**Recall ^b^**	**Precision ^c^**	**Specificity ^d^**	**Negative Predictive Value ^e^**
**Final ^f ^(Good vs Poor)**	86%	91%	90%	70%	70%
**INRG risk factors ^g ^(Good vs Poor)**	82%	85%	92%	70%	53%
**INRG Pretreatment Schema ^h ^(VLR/LR/IR vs HR)**	84%	83%	97%	80%	56%

^a ^Accuracy is the fraction of correctly classified patients and overall classified patients.

^b ^Recall is the fraction of correctly classified good outcome patients and the overall predicted good outcome patients.

^C ^Precision is the fraction of correctly classified good outcome patients and the predicted good outcome patients.

^d ^Specificity is the fraction of correctly classified poor outcome patients and the overall poor outcome patients.

^e ^Negative predictive value is the fraction of correctly classified poor outcome patients and the overall predicted poor outcome patients.

^f ^Final classifier indicates the classifier in Table 4 obtained utilizing Rulex.

^g ^INRG risk factors classifier indicates the classifier in Table 2 obtained utilizing Rulex.

^h ^INRG Pretreatment Schema classifier indicates the INRG pretreatment classification schema.

VLR = Very Low Risk; HR = High Risk; IR = Intermediate Risk; LR = Low Risk. We computed the performance of INRG pretreatment schema classifier on the 51 patients' cohort as follows.

We assigned Very low, Low, Intermediate, High risk utilizing the INRG consensus pretreatment classification schema considering only the Age at diagnosis, INSS stage and MYCN status risk factors.

We then created two groups to make the risk assignment comparable with outcome.

The first included Very low, Low and Intermediate risk patients and the second included High risk patients.

The first group was associated with a favorable outcome and the second was associated with an unfavourable outcome.

The performance of the classifier obtained considering only MYCN, age and INSS stage without the contribution of NB-hypo (INRG risk factors) (Table 2) on the 51 patients' dataset was then measured and the results are shown in Table 5. Accuracy, precision, specificity were remarkably similar. The inclusion of NB-hypo in the classifier increased somewhat recall and negative predictive value indicating an improvement of the classification of good outcome patients and better precision in predicting poor outcome patients.

Finally, we compared the Final classifier with that of the INRG consensus pretreatment classification schema on the 51 patients' dataset. To attempt this comparison we had to classify the patients in risk groups using only 3 (MYCN, age and INSS stage) out of more than 16 parameters considered by INRG and to merge Low, Very low and Intermediate risk patients into one group to have a binary classification. The results are shown in Table 5. Comparing the Final classifier with the INRG classification schema, we observed similar accuracy but differences in the other classification parameters. The Final classifier demonstrated an improvement in the classification of good outcome patients (recall: 91% vs 83%) and a better precision in predicting poor outcome patients (negative predictive value: 70% vs 56%), while INRG classification schema demonstrated a better classification of poor outcome patients (specificity: 80% vs 70%) and a better precision in predicting good outcome patients (precision: 97% vs 90% ). However, these considerations provide an indication of the relationship among these classifiers but they will have to be validated on a larger dataset.

**Figure 7:**
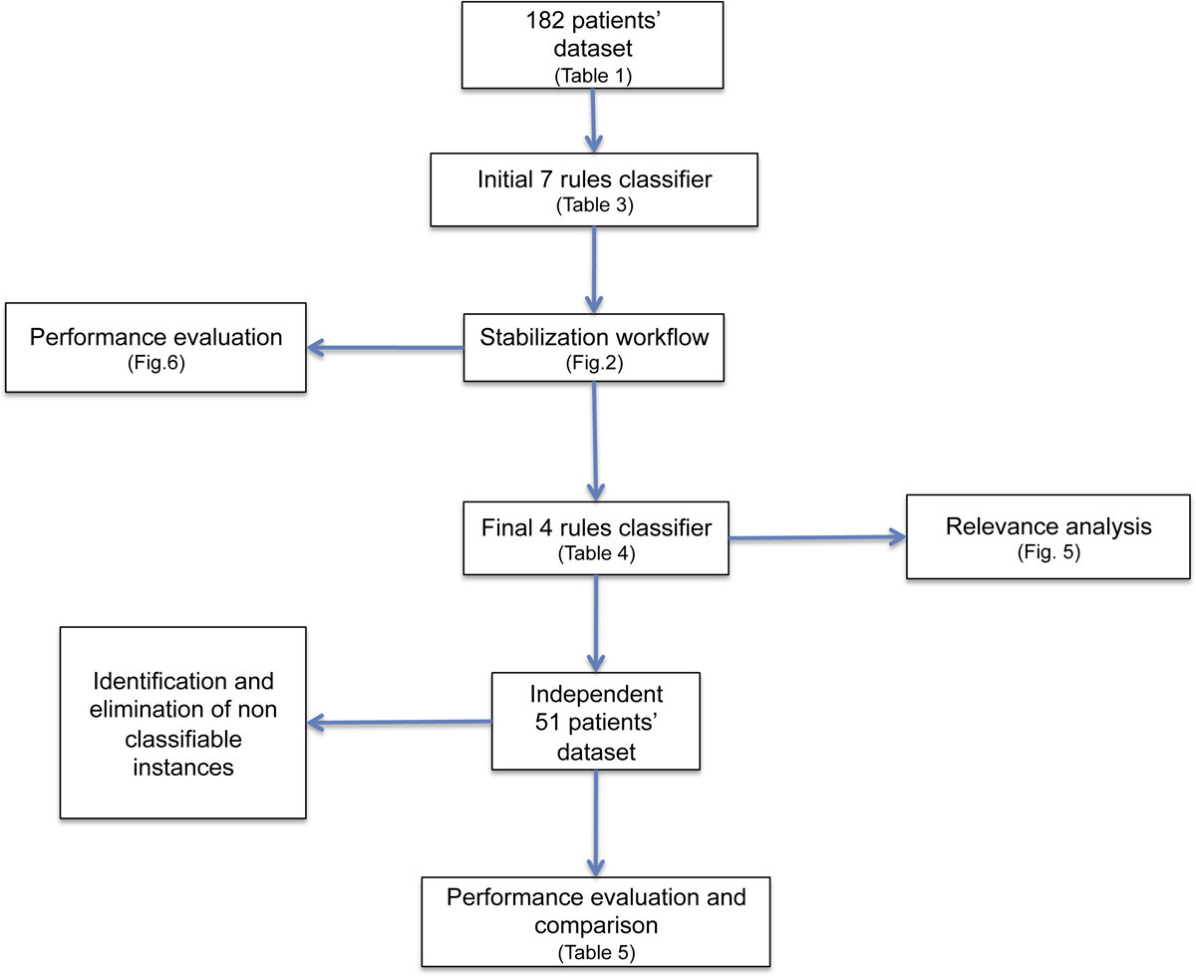
**Summing up of the rules generation**. The 182 patients' dataset was utilized to generate the initial classifier (7 rules). Following the stabilization procedure, we identified and removed the instances causing instability in the dataset to obtain a Final classifier (4 rules). The validity of the rules was analyzed by cross validation and by testing on an independent 51 patients' dataset.

In conclusion, iterative application of stabilization procedure generates fully stable rules that are a very powerful tool to classify NB-patients. Figure 7 shows the workflow of the main steps performed in our rule generation and the references to the relevant tables and figures. The stability of the rule is achieved at the expenses of including every patient in the analysis, an accepted and common practice when deciding on the rules for recruiting patients in a clinical trials.

## Discussion

We developed explicit rules, predicting the outcome of neuroblastoma patients on the bases of Age at diagnosis, MYCN amplification, INSS stage and NB-hypo signature. We demonstrate that LLM algorithms generate clinically relevant rules and that stabilization of these rules through a novel procedure, improves the performance stratifying high risk patients traditionally difficult to classify. Furthermore, the use of NB-hypo, a biology driven risk factor, identifies a cohort of poor prognosis patients as potential target of new therapeutic approaches perhaps aiming at counteracting tissue hypoxia.

Classification is central to stratification of cancer patients into risk groups benefiting of defined therapeutic approaches. Several statistical and machine learning techniques have been proposed to deal with this issue [23]. Each of them trains a specific model that is used to predict the output for new unlabeled data. We privileged classification methods capable of constructing models described by a set of intelligible rules for their immediate translation in the clinical setting and for the possibility to incorporate previous medical knowledge in the algorithm. Most available techniques, such as linear discriminant approaches, multilayer perceptrons or support vector machines, are able to achieve a good degree of provisional accuracy, but construct black-box model whose functioning cannot allow to derive information about the pathology of interest and its relationships with the considered diagnostic and prognostic factors. Different classification methods capable of constructing models described by a set of intelligible rules (if< premise> then< consequence>) were developed to overcome this drawback.

Rule generation techniques produce not only the subset of variables actually correlated with the pathology of interest, but also explicit intelligible conditions that determine a specific diagnosis/prognosis. As a consequence, relevant thresholds for each input variable are identified, which represent valuable information for understanding the phenomenon at hand. In general, each rule refers to a specific target output class and it is characterized by two statistical measures: the covering, which accounts for the fraction of examples in the training set that verify the rule and belong to the target class, and the error, which is given by the portion of patterns in the training set that satisfy the rule and do not belong to the target class. We introduced a third statistical measure, the stability, as a prerequisite for acceptance of a set of rules.

Most used rule generation techniques belong to two broad paradigms: decision trees and methods based on Boolean Function Synthesis. The approach adopted by the first kind of algorithms divides iteratively the training set into smaller subsets according to a divide and conquer strategy: this gives rise to a tree structure from which an intelligible set of rules can be easily retrieved. At each iteration a portion of the training set is split into two or more subsets according to the results of a test on a specific selected input variable. The target is to obtain at the leaves of the tree structure portions of the training set belonging to the same output class. These portions of training set are, in general, non-overlapping [28]. Decision tree methods usually provide simple rules, which can be directly interpreted by an expert, and require a reduced amount of computational resources. However, the accuracy of the developed models is often poor when compared with that exhibited by black-box techniques. The divide and conquer strategy leads to conditions and rules that point out the differences among examples of the training set belonging to different output classes. It follows that decision tree approach implements a discriminant policy: differences between output classes are the driver for the construction of the model. Several reports emphasized the unstable characteristic of the decision tree, quantifying the stability of a classifier or algorithm using syntactic or semantic stability notions [29-34].

In contrast, methods based on Boolean function synthesis adopt an aggregative policy: at any iteration some patterns belonging to the same output class are clustered to produce an intelligible rule. Suitable heuristic algorithms [24-26] are employed to generate rules exhibiting the highest covering and the lowest error; a tradeoff between these two different objectives has been obtained by applying the Shadow Clustering (SC) technique [24] which leads to final models, called Logic Learning Machines (LLM), exhibiting a good accuracy. The aggregative policy allows to retrieve intelligible rules that better characterize each output class with respect to approaches following the divide-and-conquer strategy. Clustering examples of the same kind permit to extract knowledge regarding similarities about the members of a given class rather than information about their differences. This is very useful in most applications and leads to models showing a higher generalization ability, as pointed out by intensive trials performed on different datasets through SC [35,36].

LLM is an efficient implementation of the Switching Neural Network (SNN) model [37] trained through an optimized version of the SC algorithm. LLM, SNN and SC have been successfully used in different applications: from reliability evaluation of complex systems [38] to prediction of social phenomena [39], form bulk electric assessment [40] to analysis of biomedical data [35,36,41,42]. In particular, in this last field the ability of generating models described by intelligible rules have carried out many advantages, allowing to extract important knowledge from available data. Identification of prognostic factors in tumor diseases [41] as well as selection of relevant features microarray experiments [35] are only two of the valuable targets achieved through the application of LLM and SNN. The learning approach implemented by LLM privileges the covering but this is not the unique possible approach. In the present work we privilege stability over covering and we identify a procedure to reach this aim.

Application of LLM to NB patients dataset containing the common risk factors MYCN, age at diagnosis, and INSS stage, generated rules that fit with the INRG risk assessment demonstrating the concordance of the results of LLM with previous clinical knowledge of neuroblastoma. We then included NB-hypo attribute to the classification algorithm and we demonstrated that LLM considered this risk factor relevant for outcome prediction. This result was important because it showed that NB-hypo could generate clinically relevant rules identifying previously unrecognized homogeneous groups of patients.

Clinical decisions require stability of the rules because there must be a reasonable confidence that the model is consistent and that the predictions will be insensitive to small changes in the dataset. For this reason we decided to adopt suitable methods to improve the stability of the rules even at the expenses of coverage. To this end, we developed a workflow to skim the dataset of unstable instances maximizing the stability of each rule. The stabilization procedure is iterative and it was terminated only when each rule has reached the maximal stability. The approach depends on the existence of an overlap among rules and it applies only to situations in which examples can be covered by more than one rule. This excludes the application of this kind of analysis to the decision tree representation that fragments the dataset into disjunctive subsets.

Iterative rules stabilization generated the Final classifier with four rules each endowed with the maximal stability of one. The Final classifier predicts with 3% error that NB-Hypo High, Stage 2,3,4, older that 1 year patients have poor outcome prediction (Table 4 Rule 4.2). Stage 2, 3, 4 patients older than 1 year are currently classified as either low, intermediate and high INRG risk and follow different therapeutic protocols. Our results indicate that evaluation of the NB-hypo profile can lump some of these patients into a single risk group characterized by highly hypoxic tumors therefore, candidate for new hypoxia targeted treatments substituting the current inefficient therapies.

NB-hypo and INSS stage had similar relevance in the generation of rules. INSS a critical parameter in neuroblastoma research and our data indicate that that NB-hypo is an emerging risk factor in determining the outcome. However, neither INSS nor NB-hypo reach the relevance value of 1 pointing to the need of multiple risk factors for classification.

The possibility to blend previous clinical knowledge of neuroblastoma disease with newly discovered prognostic signatures was central to the choice of the algorithm. The relevance of merging gene expression profiling, histopathological classification was reported [12,43-47]. Several studies deal with merging of genomic signatures, gene expression profiles, gene mutation, genomic instability, histopathologic and clinical classification systems in various combination for cancer classification, as exemplified by some publications [43,46,48,49]. The characterization of the tumor at diagnosis is indispensable for deciding the treatment and the NB-hypo may indicate the tumors that, as a result to the hypoxic status, express high genetic instability [50] contain undifferentiated or cancer stem cells [16,51] or a higher metastatic potential [17]. These characteristics of the primary tumor may be those that initiated the aggressiveness of the disease and could be targeted by individualized treatment. Many therapeutic agents are being developed to target hypoxia (for review see [52]) or cells in a hypoxic environment with gene therapy [53-55] and are being tested in the clinic.

The acquired stability of the rules traded off a reduced coverage causing the exclusion of 28 out of 182 patients from the dataset. This process is similar to that used to design clinical protocols that is based upon selective recruitment of a narrow group of patients with similar expression of risk factors. There were obvious reasons to exclude some patients from the database. Twenty four instances shared the same attributes but were equally distributed in the two outcome classes. Under such conditions the classification was entirely dependent upon the casual imbalance of the distribution of such patients in the training set. Similar consideration applied to the other patients that were excluded from the dataset to improve the stability of the rules. We conclude that our system generated a third class of non classifiable patients. From a clinical perspective, there is no loss in excluding these patients from the classification because no significant prediction could be made by this or any other algorithm on such an ambiguous cohort. In contrast, a clear gain is achieved by strengthening the stability of the rules that cover the remaining 85% of the patients. We identified instances that caused instability on our dataset but we do not exclude that others possible causes of instability could emerge in other situations and studies are in progress to address these issues.

Removal of the instances that caused instability was instrumental to prevent theoretical conflicts in the classification. Superficial inspection of the Final classifier leads to the conclusion that there may one group of patient characterized by opposite prediction. However, such instances do not occur in the dataset after removal of the instability cases. Therefore it is mandatory that the process of generating the rules of the Final classifier be coupled with the stability analysis and removal of instability generating instances. It is noteworthy that instability is not limited to potentially conflicting instances but to other situations including a very low covering. In this case, the casual partition of such instances between training and test sets is the true gauge for outcome prediction.

Rulex can be tuned to perform in such a way that it associates every instance with a prediction. Under such constrains it deals with conflicting situation by favoring the rule that has the maximal covering among the various possibilities. We did not feel that the covering evaluation was a sufficient indicator to classify patients outcome. For this reason we preferred to choose the stabilization workflow to identify the truly stable rules.

The performance of the Final classifier was validated in an independent dataset to exclude the danger of overfitting caused by the removal of instability instanced and testing in cross validation in the purged dataset. The performance statistical measures in the test set were quite satisfactory demonstrating the efficacy of the classifier in an unrelated dataset. The values were somewhat lower than those observed in cross validation. Interestingly, the percentage of instances generating instability were the same in both 182 and 51 patients' datasets. These performance statistical measures are comparable, but somewhat lower to those obtained in cross validation. The relatively small number of poor outcome patients in the testing set may account for the reduced specificity observed. A larger testing data set would be needed to obtain a more accurate assessment of the performance.

## Conclusions

The novelty of our work is to target stability, explicit rules and blending of risk factors as the characterizing elements to generate classification rules for NB patients to be conveyed to the clinic and to be used to design new therapies.

Our first aim was to explore algorithms that could generate intelligible classification rules of neuroblastoma patients easily translatable into the clinical setting. We found that LLM implemented by Rulex, was a reliable algorithm, generating rules that paralleled the risk stratification of NB patients obtained by clinical previous knowledge.

The second major task was to develop a prognostic classifier of NB outcome capable to blend existing clinical and molecular risk factors derived from previous clinical knowledge of the disease with the newly discovered prognostic NB-hypo signature assessing the hypoxic status of the neuroblastoma tumor. We found that NB-hypo could be successfully associated to other known risk factors to generate relevant prognostic rules capable to stratify high risk patients.

We identified stability as a critical factor in the translation of the classifier into clinic and we developed a framework to maximize the stability of the rules at the expenses of the coverage. The final product was a very stable classifier covering 85% of the patients comprising the original dataset. This procedure can be easily extended to other classifiers provided that the instances are covered by more than one rule.

From the biology standpoint we found that NB-hypo is an important risk factor for neuroblastoma patients that can help to resolve high risk stage 4 patients as well as those with good prognosis. We propose that the Final classifier derived in the present work be utilized as bases for designing new therapies needed for the poor prognosis patients correctly included in the NB-hypo containing rules of our Final classifier.

## Methods

### Patients

The rules were established in a 182 neuroblastoma patients' dataset belonging to four independent cohorts that were enrolled on the bases of the availability of gene expression profile by Affymetrix GeneChip HG-U133plus2.0 and clinical and molecular information. Eighty-eight patients were collected by the Academic Medical Center (AMC; Amsterdam, Netherlands) [22,56]; 21 patients were collected by the University Children's Hospital, Essen, Germany and were treated according to the German Neuroblastoma trials, either NB97 or NB2004; 51 patients were collected at Hiroshima University Hospital or affiliated hospitals and were treated according to the Japanese neuroblastoma protocols [57]; 22 patients were collected at Gaslini Institute(Genoa, Italy) and were treated according to Italian AIEOP or European SIOPEN protocols. The data are stored in the R2: microarray analysis and visualization platform [58] (AMC and Essen patients) or at the BIT-neuroblastoma Biobank of the Gaslini Institute. The investigators who deposited the data in the R2 repository agree to use the data for this work. In addition, we utilized the data present on the public database at the Gene Expression Omnibus number GSE16237) for Hiroshima patients [57]. Informed consent was obtained in accordance with institutional policies in use in each country. In every dataset, median follow-up was longer than 5 years and tumor stage was defined as stages 1, 2, 3, 4, or 4s according to the International Neuroblastoma Staging System (INSS). The main features of the 182 neuroblastoma patients are listed in Table 1. Good and poor outcome were defined as the patient's status alive or dead 5 years after diagnosis. The pre-treatment risk groups of the 182 patients were assigned according to the International Neuroblastoma Risk Group (INRG) Consensus Pretreatment Classification schema [59]. We utilized an independent 51 patients dataset collected at the Gaslini Institute (Genoa, Italy) treated according to the Italian AIEOP protocol to investigate the predictive accuracy of the algorithm. Good and poor outcome were defined as the patient's status alive or dead 18 months after diagnosis.

### Gene expression analysis

Gene expression profiles for the 182 tumors were obtained by microarray experiment using Affymetrix GeneChip HG-U133plus2.0 and the data were processed by MAS5.0 software according Affymetrix's guideline.

### Unsupervised cluster analysis

182 NB patients' cohort was clustered by k-means analysis of the 62 probe sets constituting the NB-hypo signature previously described to measure tumor hypoxia [22]. The Sum of within cluster distance was calculated to establish the optimal partition (see Additional file 1). Analysis was performed using 500 iterations, preserving instances order and using Manhattan distance implemented in the WEKA package [60].

### Rules generation

In a *classification *problem *d*-dimensional examples $x\in {ℜ}^{d}$ are to be assigned to one of *q *possible classes, labeled by the values of a categorical output *y*. Starting from a *training set S *including *n *pairs (***x***_*i*_,*y_i_*), *i *= 1,..., *n*, deriving from previous observation, techniques for solving classification problems have the aim of generating a model *g*(***x***), called *classifier*, that provides the correct answer *y *= *g*(***x***) for most input patterns ***x***. Concerning the components *x_j _*two different situations can be devised:

1 **ordered variables**: *x_j _*varies within an interval [*a,b*] of the real axis and an ordering relationship exists between its values.

2 **nominal (categorical) variables**: *x_j _*can assume only the values contained in a finite set and there is no ordering relationship among them.

*Rule generation methods *constitute a class of classification techniques that generate intelligible models *g*(***x***) described by a set of *m *rules *r_k_, k *= 1,..., *m*, in the **if-then **form:

if<premise>then<consequence>

where <*premise*> is the logical product (and) of *m_k _*conditions *c_kl_*, with *l*= 1,..., *m_k_*, on the components *x_j_*, whereas <*consequence*> gives a class assignment $y=\tilde{y}$ for the output. In general, a condition *c_kl _*in the premise involving an ordered variable *x_j _*has one of the following forms *x_j _*>*λ, x_j _*≤ *μ, λ *<*x_j _*≤ *μ*, being *λ *and *μ *two real values, whereas a nominal variable *x_k _*leads to membership conditions *x_k _*∈ {*α, δ, σ*}, being *α, δ, σ *admissible values for the *k*-th component of ***x***.

For instance, if *x*1 is an ordered variable in the domain {1,..., 100} and *x*_2 _is a nominal

component assuming values in the set {*red, green, blue*}, a possible rule *r*_1 _is

$$if\;x_{\text{1}}\;>\;\text{4}0\;and\;x_{\text{2}}\in \left\{red,blue\right\}\;then\;y\;=\;0$$

where 0 denotes one of the *q *possible assignments (classes).

According to the output value included in their consequence part, the *m *rules *r_k _*describing a given model *g*(***x***) can be subdivided into *q *groups *G*_1_, *G*_2_,..., *G_q_*. Considering the training set *S*, any rule *r *∈ *G_l _*is characterized by four quantities: the numbers *TP*(*r*) and *FP*(*r*) of examples (***x***_*i*_,*y_i_*) with *y_i _*= *y_l _*and *y_i _*≠ *y_l_*, respectively, that satisfy all the conditions in the premise of *r*, and the numbers *FN*(*r*) and *TN*(*r*) of examples (***x***_*i*_,*y_i_*) with *y_i _*= *y_l _*and *y_i _*≠ *y_l_*, respectively, that do not satisfy at least one of the conditions in the premise of *r*. Starting from *TP, FP, TN*, and *FN*, other useful characteristic quantities, such as the *covering C*(*r*), the *error E*(*r*), and the *precision P*(*r*) can be derived:

$$C\left(r\right)=\frac{TP\left(r\right)}{TP\left(r\right)+FN\left(r\right)},\;E\left(r\right)=\frac{FP\left(r\right)}{TN\left(r\right)+FP\left(r\right)},\;P\left(r\right)=\frac{TP\left(r\right)}{TP\left(r\right)+FP\left(r\right)}$$

*C*(*r*) and *P*(*r*) are usually adopted as measures of relevance for a rule *r*; as a matter of fact, the greater is the covering and the precision, the higher is the generality and the correctness of the corresponding rule.

On the other hand, to obtain a measure of relevance *R*(*c*) for a condition *c *included in the premise part of a rule *r*, one can consider the rule *r*' obtained by removing that condition from

*r*. Since the premise part of *r*' is less stringent, we obtain that *E*(*r*') ≥ *E*(*r*) so that the difference *R*(*c*) = *E*(*r*')-*E*(*r*) can be used as a measure of relevance for the condition *c *of interest. Another possible choice is given by *R*(*c*) = *P*(*r*)-*P*(*r*'), but in this case we can obtain negative values of relevance.

Since each condition *c *refers to a specific component of ***x***, it is also possible to define a measure of relevance *R_j _*for every input variable *x_j_*:

$$R_{j}=1-\underset{K}{\prod }\left(1-P\left(r_{{}_{k}}\right)R\left(c_{kl}\right)\right)$$

where the product is computed on the rules *r_k _*that includes a condition *c_kl _*on the variable *x_j_*.

The relevance of a variable *x_j _*depends on the precision *P*(*r_k_*) of the rules *r_k _*containing a condition *c_kl _*of that variable and of the margin *R*(*c_kl_*) of the classification error in the training set introduced by the condition *c_kl _*. Therefore, the relevance increases with the magnitude of the precision of the rules that include the variable and with the margin of the classification error introduced by a specific condition. The relevance can have only values between 0 and 1 because the precision and error values range from 0 and 1 and so it is their product. The relevance can be computed for the entire dataset and for each class. In the latter case, only the rules predicting the expected class are selected.

One of the rule generation methods is Logic Learning Machine (LLM), an efficient implementation of the Switching Neural Network (SNN) model [37] trained through an optimized version of the Shadow Clustering (SC) algorithm [24]. By applying LLM it is possible to derive a set of intelligible rules possessing a generalization ability comparable and even superior to that of best machine learning techniques, maintaining the possibility of understanding the mechanism involved in the classification process.

The LLM is implemented by the *Rulex *software suite [61]. The Rulex software, developed and commercialized by Impara srl, is an integrated suite for extracting knowledge from data through statistical and machine learning techniques. An intuitive graphical interface allows to easily apply standard and advanced algorithms for analyzing any dataset of interest, providing the solution to classification, regression and clustering problems.

The model *g*(***x***) generated by the LLM task of Rulex can be utilized to produce the output class for any input pattern x^*^. The <*premise*> part of each of the *m *intelligible rules *r_k_, k *= 1,..., *m*, describing the model *g*(***x***), is checked to determine whether it is verified by a given sample x^*^. If only one rule *r_k _*is satisfied by x^*^_' _then the <*consequence*> part of *r_k _*will provide the class y = y^* ^to be assigned to x^*^. In contrast, if the <*premise*> part of two or more rules *r_k _*is verified by x^*^, Rulex will choose the class included in the <*consequence*> part of the rule with the highest covering value *C*(*r_k_*). Once we obtained our final classifier *g*(***x***), including *m *intelligible rules *r_k_, k *= 1,..., *m*, we can check whether conflicting rules are present in *g*(***x***), i.e. rules that provide different outputs corresponding to the same pattern ***x***. Be *y_k _*the output value included in the <*consequence*> part of the *k*th rule, two rules *r_h _*and *r_k _*are *conflicting *if both of them are verified by a same pattern ***x ***and *y_h _*≠ *y_k_*.

To build classifiers we used a number of graphical components provided by Rulex 2.0. We utilized Visualization\editing components to visualize and export the confusion matrix, the training and validation sets, the rules of the classifier, to access statistical data (e.g. Covering, Error and Relevance) and to edit the attributes. We utilized a discretizer component with attribute driven incremental discretization as method for discretization and minimum distance between different classes of 20% to pre-process the data. We utilized the Logic Learning Machine classification component building rules in bottom-up-mode, minimizing number of conditions, allowing to exceed maximum number of conditions and having maximum error allowed on the training set of 0%. By setting upper maximum error allowed on the training set to 0% we forced LLM to generate rules that cover every instance of the training set.

### Stabilization procedure

To assess stability of the classifiers generated by Rulex we developed an independent procedure called *Stabilization*. The idea of the stabilization is to calculate the degree of stability of a classifier generated by Rulex and perform a number of suitable transformations of the dataset in such a way that following generations of classifiers could be more stable than the preceding one. The process stops when a classifier generated stable results. The workflow of the stabilization procedure is shown in Figure 2.

The procedure starts by our initial 182 patients' dataset. We execute LLM on the overall dataset and we generated initial classifications rules. We then perform 5 independent 10-fold cross validations executing LLM for each one of the 50 iterations. We obtain 50 classifiers trained in different training sets and perform an analysis of stability by calculating a stability value for each rule of the temporary classifier. Given two classifiers *g*(***x***) and *g'*(***x***) generated in two distinct iterations of *n *independent *m*-fold cross validations, we say that r_k _and *r_h _are occurrences *of the same rule *r *if and only if for some distance metric *d, d(r_k_, r,)<*ε and *d(r, r_h_)<*ε for some arbitrary value ε. From the occurrences we define a new statistical measure called *stability *as follows. Given a classifier *g*(***x***), a rule *r_k _*∈ *g*(***x***) *and *a number *n+m *of classifiers obtained by *n *independent *m*-fold cross validations, we say that *Stability(r_k_) = b/(n+m) for some 0 *≤ *b *≤ *n+m *if and only if exist *b *occurrences of *r_k _*in the *n+m *classifiers. Given a classifier *g*(***x***), we say that *g*(***x***) is a *stable classifier *if and only if for each rule *r_k _*∈ *g*(***x***) we have (1-ν*) *≤ *Stability(r_k_) *≤ *1 for some *ν <1 arbitrarily selected. The maximum stability of a rule is when *Stability(r_k_) = 1*. We compute the stability for every classification rule. If each of them is stable we stop the stabilization procedure, and the classification rules become the Final classifier. If even one rule is not stable, we identify the instances in the initial dataset that are the causes of the instability. To this end we introduce the notion of *Core rules*. Given two classifiers *g*(***x***) and *g*'(***x***) and two rules *r_k _*=(***x***_*i*_,*y_i_*) ∈ *g*(***x***) and *r_h _*=(***x***_*j*_,*y_i_*) ∈ *g*'(***x***), we define a *Core rule *r a rule of the form r = (***x***_*i *_∩ ***x***_*j*_, *y_i_*). Core rule is a rule obtained by intersecting the conditions of two or more rules generated by distinct classifiers. A Core rule gives information about what instances remained conserved through cross validations and we use it to identify instances that do not preserve through cross validations.

We developed a procedure, named "CORE procedure" to identify the Core rules. Given sets of rules generated in a number *n+m *iterations of cross validations, the CORE procedure performs the following steps. First, it identifies similar rules from the *n+m *classifiers. Second, it generates a Core rule from these rules. Third, it calculates the stability statistical measure of the Core rule from the stability statistical measures of the component rules. Fourth, it returns the Core rule set and the stability values.

We utilize the information given by the stability statistical measure and the Core rules to identify the cause of instability among the instances of the dataset. A number of situations can generate instability in the rules of a classifier generated with LLM. We identified three major specific causes of instability. The first regards instances covered by a given rule *r_k _*for which *Stability(r_k_)<1-*ν *and Covering(r_k_)*≤ *s *for some real number *s<1*. In this case, if *s *is small (e.g. *s = 0.1 *) LLM could generate a rule that covers the instances only when all of them are in the training set, but LLM will not generate that rule when one or some them is in the validation set. To introduce the second cause of instability we need to extend our notation. Given a rules *rk *∈ *g*(***x***) we introduce the set H(*r_k_*) of the instances of the training set that are covered by *r_k_*.. Given two rules *r_k _, r_h _*∈ *g*(***x***) such that H(*r_k_*) \ H(*r_h_*) = H(r') where | H(r') |<s for some small natural number *s*≥ *1 *(e.g. *s = 1*), the instances in H(r') can be the second cause of instability. In this case, LLM generates both rules in the same classifier only when the instances in H(r') are in the training set. The third cause of instability could occur when in the dataset there is a group of instances with the same premises but different consequences (i.e. same premise but half of them has a class value different from the other half). In this case, LLM generates different rule set if in the training set there is a larger number of instances of one class or of another. The instances identified as cause of instability are then removed from the dataset and the purged new dataset is used as new input dataset.

### Statistical analysis

To test the statistical significance of the rules we used a Fisher's Exact test (FET) implemented by the software package R. Given a rule *r:(X,y)*, FET calculates the exact probability of observing the particular arrangement of the number of instances satisfying × and y, × and ¬y, ¬X and y and ¬X and ¬y assuming the marginal total y and ¬y under the null hypothesis that *X *and ¬*X *are equally probable to have y as consequent. We define y as good outcome patients and ¬y as poor outcome when we considers rules classifying good outcome. We switch y with ¬y for the rules classifying poor outcome patients.

To test the predictions of the classifiers we use the following metrics: accuracy, recall, specificity, negative predictive values (NPV) and we considered good outcome patients as positive instances and poor outcome patients as negative instances. Accuracy is the proportion of correctly predicted in the overall number of instances. Recall is the proportion of correctly positive predicted against all positive of the dataset. Precision is the proportion of the positives correctly classified against all the predicted positive. Specificity is the proportion of correctly negative predicted against all the negative of the dataset. NPV is the proportion of the negative correctly classified against all the predicted negative.

To summarize and display the distribution of the performance we utilized boxplot diagrams. Boxplot shows a box that contains the 50% of the dataset. The upper edge of the box indicates the 3rd quantile while the lower indicates the 1st quantile. The range in the middle between 1st-3rd quantile is known as *inter-quantile range *(IQR). The line within the box indicates the median (2^nd ^quantile). The ends of the vertical whiskers indicate the minimum and the maximum value of the dataset and when outliers are present in the dataset the whiskers extend until a maximum of 1.5 times the IQR area. Any value outside these points is considered as a potential outlier and is represented with a circle.

## List of abbreviations used

INSS: International Neuroblastoma Staging System; OS: overall survival; EFS: event free survival; FET: Fisher's Exact test; NPV: Negative Predictive Value; SIOPEN: Society of Pediatric Oncology European Neuroblastoma; INRG: International Neuroblastoma Risk Group; LLM: Logic Learning Machine; SNN: Switching Neural Networks; SC: Shadow Clustering; LR: Low Risk; IR: Intermediate Risk; HR: High Risk; TP: true positives; FP: false positives; TN: true negatives; FN: false negatives; NB: neuroblastoma; IQR: Interquantile range.

## Competing interests

The authors declare that they have no competing interests.

## Authors' contributions

DC performed computer experiments and the statistical analysis, developed the stabilization paradigm, helped to draft the manuscript. MM suggested the use of LLM, designed some of the experiments, designed the Rulex software and helped to draft the manuscript. RV, AE, AS, AG, CG participated to the development of the project. FB carried out the microarray data analysis. LV conceived the project, supervised the study and wrote the manuscript.

## Declarations

Charge for this article was paid by a grant of the Italian Association for Cancer Research (AIRC).

## Supplementary Material

Additional file 1 **Patients' clustering according to the NB-hypo signature**. the file contains two figures relative to the k-mean clustering of the 182 patients into groups differing for the expression of the 62-probe sets NB-hypo signature. Additional file 1. Figure 1. *Number of clusters selection*. NB-hypo 62 probe sets gene expression data of the 182 patients cohort were clustered using k-means algorithm and the Sum of within cluster distance was calculated. The figure shows the plot of the Sum of within cluster distance varying the initial number of clusters. The curve is basically flat after two clusters showing that the dichotomization of the dataset is preferable and that no major gain can be achieved dividing the dataset further. Additional file 1. Figure 2*. Heatmap of clustered 62 probe sets of the NB-hypo signature *The expression data for each probe set were scaled and represented by pseudo-colors in the heatmap. Red color corresponds to high level of expression and green color corresponds to low level of expression. These data confirm previous findings on a smaller number of patients [22] that k-means clustering dichotomized High and Low NB-hypo expressing tumors. Click here for file

Additional file 2 **Classifiers generated by the stabilization procedure**. Description of data: the stabilization procedure is described in the Methods section and summarized in Figure 2. The procedure calls for an iterative approach and the intermediate results are collected in the three tables of Additional file 2. Additional file 2. Table 1*Core rules from the first iteration of the stabilization procedure*. The Core rules generated by the first iteration of the stabilization procedure are shown together with prediction outcome and stability of each rule. Additional file 2. Table 2. *Classification rules of the second iteration on a purged 155 patients' dataset*. The dataset is reduced because the instances causing instability were removed in the first iteration. The classification rules generated by the second iteration of the stabilization procedure are shown together with prediction outcome and stability of each rule. Additional file 2. Table 3*Core rules from the second iteration of the stabilization procedure*. The Core rules generated by the second iteration of the stabilization procedure are shown together with prediction outcome and stability of each rule. Click here for file
