# Supplementary material for: Logic Learning Machine creates explicit and stable rules stratifying neuroblastoma patients
Source: BMC Bioinformatics. 2013 Apr 22;14(Suppl 7):S12. doi: 10.1186/1471-2105-14-S7-S12 (PMC3633028; doi:10.1186/1471-2105-14-S7-S12)

**Additional file 1**

**Fig. 1 - Number of clusters selection.**

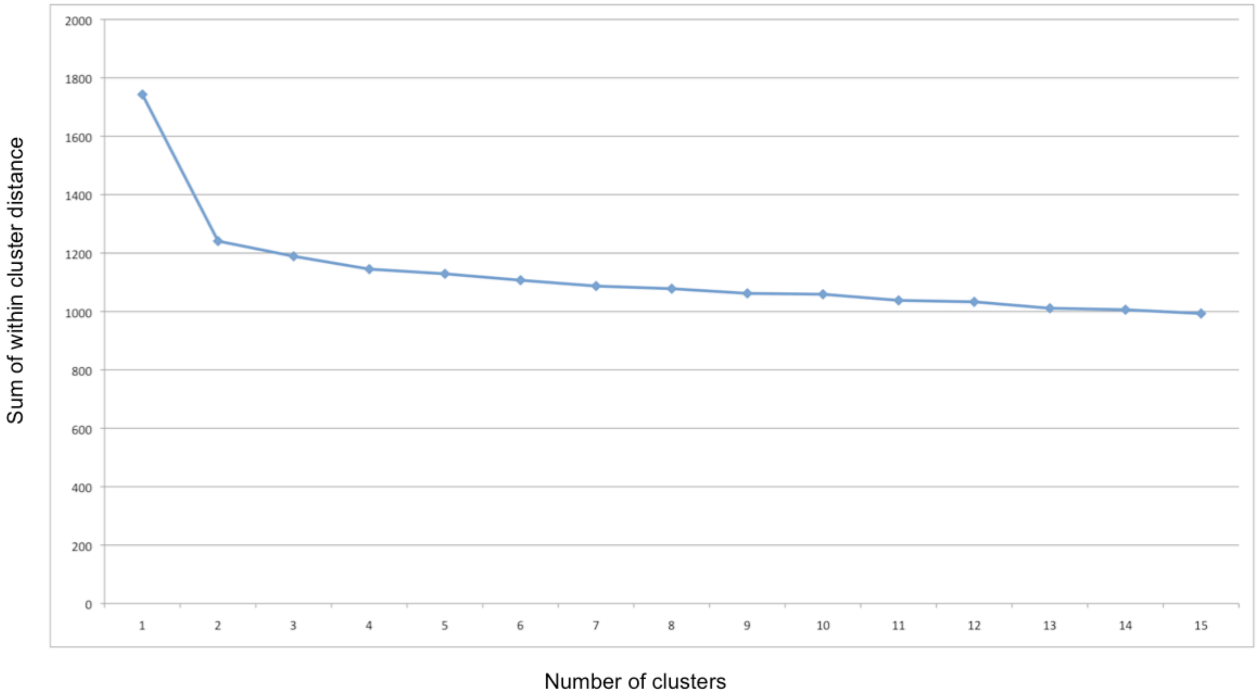

**Fig. 2 - Heatmap of clustered 62 probe sets of the NB-hypo signature**

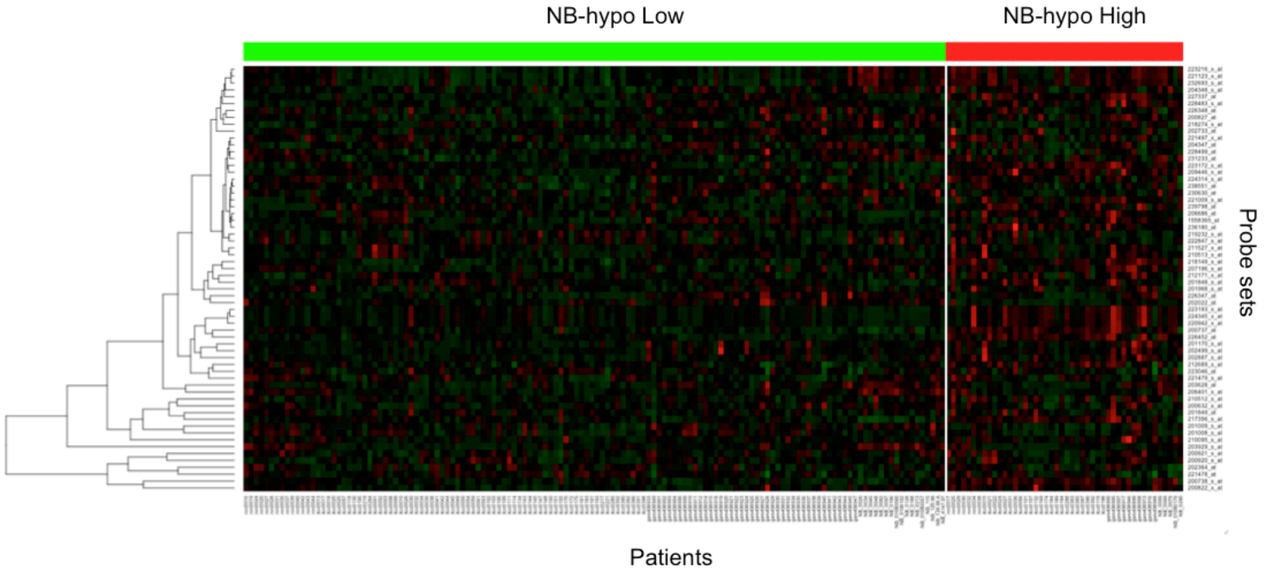

Supplement: Additional file 1 — Patients' clustering according to the NB-hypo signature. the file contains two figures relative to the k-mean clustering of the 182 patients into groups differing for the expression of the 62-probe sets NB-hypo signature. Additional file 1. Figure 1. Number of clusters selection. NB-hypo 62 probe sets gene expression data of the 182 patients cohort were clustered using k-means algorithm and the Sum of within cluster distance was calculated. The figure shows the plot of the Sum of within cluster distance varying the initial number of clusters. The curve is basically flat after two clusters showing that the dichotomization of the dataset is preferable and that no major gain can be achieved dividing the dataset further. Additional file 1. Figure 2. Heatmap of clustered 62 probe sets of the NB-hypo signature The expression data for each probe set were scaled and represented by pseudo-colors in the heatmap. Red color corresponds to high level of expression and green color corresponds to low level of expression. These data confirm previous findings on a smaller number of patients [22] that k-means clustering dichotomized High and Low NB-hypo expressing tumors. [file 1471-2105-14-S7-S12-S1.pdf]
