# Supplementary material for: Logic Learning Machine creates explicit and stable rules stratifying neuroblastoma patients
Source: BMC Bioinformatics. 2013 Apr 22;14(Suppl 7):S12. doi: 10.1186/1471-2105-14-S7-S12 (PMC3633028; doi:10.1186/1471-2105-14-S7-S12)
Supplement: Additional file 2 — Classifiers generated by the stabilization procedure. Description of data: the stabilization procedure is described in the Methods section and summarized in Figure 2. The procedure calls for an iterative approach and the intermediate results are collected in the three tables of Additional file 2. Additional file 2. Table 1Core rules from the first iteration of the stabilization procedure. The Core rules generated by the first iteration of the stabilization procedure are shown together with prediction outcome and stability of each rule. Additional file 2. Table 2. Classification rules of the second iteration on a purged 155 patients' dataset. The dataset is reduced because the instances causing instability were removed in the first iteration. The classification rules generated by the second iteration of the stabilization procedure are shown together with prediction outcome and stability of each rule. Additional file 2. Table 3Core rules from the second iteration of the stabilization procedure. The Core rules generated by the second iteration of the stabilization procedure are shown together with prediction outcome and stability of each rule. [file 1471-2105-14-S7-S12-S2.pdf]

Additional file 2

Table 1 – Core rules from the first iteration of the stabilization procedure.

Rule ID[a]		NB-hypo	INSS Stage	MYCN Status	Age at diagnosis (Years)		Predicted Outcome	Stability[b]
**A1.1**	*IF (*	_	_	_	< 1	*)THEN*	Good	0.94
**A1.2**	*IF (*	High	{2, 3, 4}	normal	≥ 1	*)THEN*	Poor	0.8
**A1.3**	*IF (*	High	{3, 4}	_	≥ 1	*)THEN*	Poor	1
**A1.4**	*IF (*	Low	{1, 2, 3, 4s}	normal	_	*)THEN*	Good	1
**A1.5**	*IF (*	_	{1, 2, 4s}	amplified	_	*)THEN*	Good	0.8
**A1.6**	*IF (*	_	{1, 4s}	normal	_	*)THEN*	Good	1
**A1.7**	*IF (*	_	{4}	amplified	≥ 1	*)THEN*	Poor	1

[a] The Rule ID is composed by the A (additional file), followed by the table number, a dot and the rule number.

[b] Stability is the fraction of the occurrences of a given rule in a 5 rounds of 10 fold cross validations.

Table 2 – Classification rules of the second iteration on a purged 155 patients' dataset

Rule ID[a]		NB-hypo	INSS Stage	MYCN Status	Age at diagnosis (Years)		Predicted Outcome	Covering[b] (%)	Error[c] (%)	Fisher pvalue[d]	Stabilty[e]
**A2.1**	*IF (*	_	{4}	_	≥ 1	*)THEN*	Poor	89	4	<0.001	1
**A2.2**	*IF (*	High	{2, 3, 4}	_	≥ 1	*)THEN*	Poor	86	3	<0.001	1
**A2.3**	*IF (*	Low	_	normal	_	*)THEN*	Good	89	0	<0.001	1
**A2.4**	*IF (*	_	{1, 2, 4s}	_	_	*)THEN*	Good	74	0	<0.001	0.9
**A2.5**	*IF (*	_	_	_	< 1	*)THEN*	Good	72	0	<0.001	0.88

[a] The Rule ID is composed by the A that indicate a rule in an additional file, followed by the table number, a dot and the rule number.

[b] Covering is the fraction of examples in the training set that verify the rule and belong to the target class.

[c] Error is the fraction of examples in the training set that satisfy the rule and do not belong to the target class.

[d] Fisher p-value quantifies the statistical significance of the rule.

[e] Stability measures the fraction of the occurrences of a given rule in a 5 rounds of 10 fold cross validations.

Table 3 – Core rules from the second iteration of the stabilization procedure.

Rule ID[a]		NB-hypo	INSS Stage	MYCN Status	Age at diagnosis (Years)		Predicted Outcome	Stability[b]
**A3.5**	*IF (*	_	{4}	_	≥ 1	*)THEN*	Poor	1
**A3.2**	*IF (*	High	{2, 3, 4}	_	≥ 1	*)THEN*	Poor	1
**A3.3**	*IF (*	Low	_	normal	_	*)THEN*	Good	1
**A3.4**	*IF (*	_	{1, 2, 4s}	normal	_	*)THEN*	Good	1
**A3.1**	*IF (*	_	_	_	< 1	*)THEN*	Good	0.78

[a] The Rule ID is composed by the A (additional file), followed by the table number, a dot and the rule number.

[b] Stability is the fraction of the occurrences of a given rule in a 5 rounds of 10 fold cross validations.
